# A long-term photographic dataset for individual identification of the Balearic wall lizard

**DOI:** 10.1038/s41597-026-07411-z

**Published:** 2026-05-13

**Authors:** Roberto Alcaraz, Balma Albalat-Oliver, Alejandro Villa, Giacomo Tavecchia, José Manuel Igual, Andreu Rotger

**Affiliations:** 1 Mediterranean Institute for Advanced Studies (IMEDEA, UIB–CSIC), Esporles, 07190 Spain; 2 Animal Demography and Ecology Unit, IMEDEA (CSIC–UIB), Esporles, 07190 Spain; 3 Department of Biology, University of the Balearic Islands, Palma, 07122 Spain

## Abstract

We present BalearicLizard, a curated, long-term collection of high-resolution photographs of the Balearic wall lizard (*Podarcis lilfordi*) designed to support non-invasive individual re-identification, ecological monitoring and computer-vision benchmarking. The dataset comprises **4,619 images** from **1,009 individuals** acquired during 15 years of systematic capture–recapture monitoring (from October 2010 to September 2024) on Illot d’en Curt, a small islet off the southern coast of Mallorca in the Balearic Islands, Spain. For each capture, we provide the original field photograph, a derived crop focusing on the ventral scale pattern, and standardised metadata including capture date, individual identifier and file location. Individual lizards were manually identified and curated by expert observers based on distinctive ventral scale arrangements, with historical mislabellings corrected using automated re-identification models. BalearicLizard offers a high-quality benchmark for developing and evaluating individual re-identification methods in small-bodied reptiles and a reusable resource for demographic and conservation studies of endemic island lizard populations. Data are released under CC BY 4.0 via Kaggle (https://www.kaggle.com/datasets/roberalcaraz/baleariclizard).

## Background & Summary

Understanding population dynamics, survival rates and habitat use patterns is fundamental for effective conservation management, particularly for endemic insular species with restricted distributions and small population sizes. The Balearic wall lizard (*Podarcis lilfordi*) exemplifies these conservation challenges. Endemic to the Balearic Islands and structured into distinct evolutionary significant units (ESUs), this small lacertid exhibits pronounced morphological and genetic differentiation among islands^1^. Its current distribution is limited to the Cabrera archipelago and satellite islets of Mallorca and Menorca, following extirpations on the main islands driven largely by introduced predators and habitat change^2^. This narrow, fragmented distribution places the species at risk and underpins its current classification as Endangered by the IUCN^2^.

The intrinsic fragility of insular ecosystems further emphasises the need for long-term monitoring to detect environmental change and support evidence-based conservation management aimed at ensuring population persistence. Robust demographic estimates for small vertebrates are typically obtained through capture–mark–recapture (CMR) methods. However, traditional marking methods for small lizards often rely on invasive techniques such as toe-clipping, scale-branding or passive integrated transponder (PIT) tags^3^, which may affect survival, behaviour and demographic parameter estimates^4^, and require repeated handling of individuals. To address these limitations, photo-identification based on natural scale patterns provides a non-invasive alternative that minimises handling stress while allowing reliable long-term individual monitoring. In *P. lilfordi*, the configuration and shape of ventral scales constitute stable, individual-specific biometric markers that persist throughout life, making photo-identification a robust approach for demographic studies in insular lizard populations^5^. With the advent of digital photography, attention has increasingly focused on using natural marks such as spots, scale patterns or colouration for individual recognition of reptiles and other vertebrates.

Despite these advances, real-world image-based datasets from small reptiles still pose substantial technical challenges. First, assembling well-annotated image collections is difficult, and determining which photographs belong to the same individual requires meticulous and time-consuming manual verification. Second, the resulting datasets typically suffer from class imbalance, with some highly active or site-faithful individuals photographed dozens of times, while many others appear only once or twice. This phenomenon of trap-dependence can cause learning algorithms to overfit to frequently observed identities. Third, practical deployments must handle open-set scenarios, in which previously unseen individuals enter the study population over time, requiring models not only to match known subjects accurately, but also to detect and register novel individuals without inflating false-positive identification rates.

**Fig. 1:**
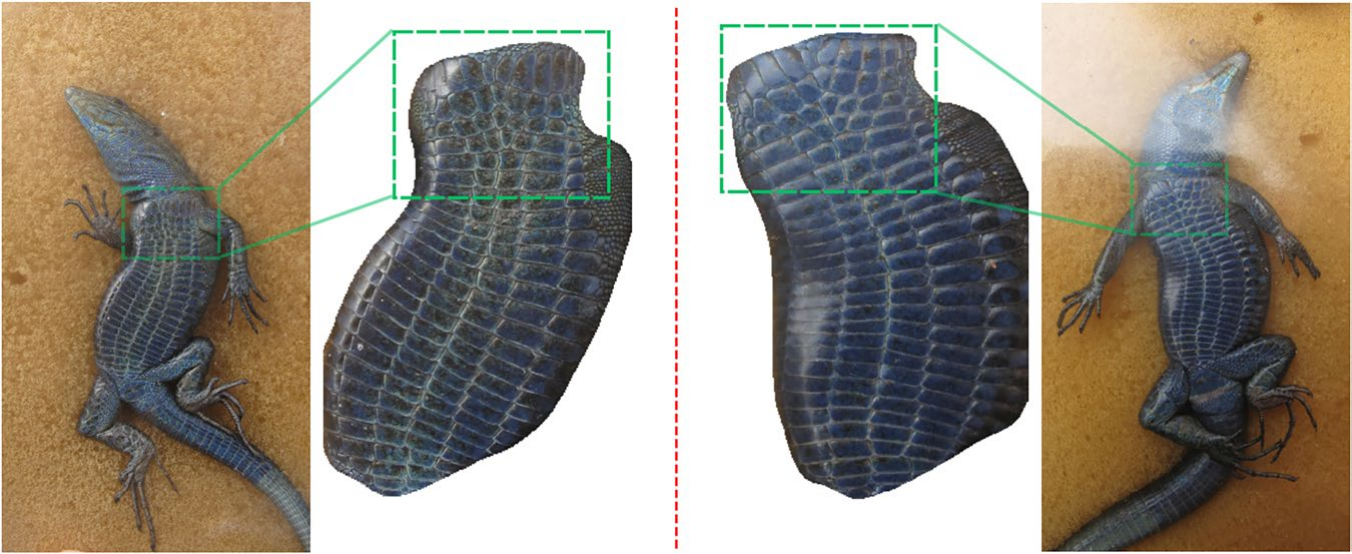
Stability of the ventral scale pattern across recaptures. Example of two photographs of the same individual (C910) taken on different sampling dates. For each capture, the full ventral photograph is shown together with the corresponding segmented body region. The insets highlight the pectoral and anterior ventral scales used as biometric markers in BalearicLizard^6^, illustrating the high stability of the pattern over time that underpins reliable photo-identification.

To address the challenges outlined above, we introduce BalearicLizard^6^, a comprehensive dataset for individual re-identification of *Podarcis lilfordi*. Figure 1 illustrates the stability of the ventral pattern across recaptures of the same individual and the correspondence between full-frame images and their segmented body regions.

### Related Work

Several public image datasets already support individual identification in reptiles and related herpetofauna, although most focus on marine turtles. The SeaTurtleID2022 dataset^7^ is the best-known example: it provides 8,729 photographs of 438 wild sea turtles collected over 13 years, with individual IDs, encounter timestamps and body-part segmentation masks, and it introduces time-aware closed- and open-set splits that have become a benchmark for long-term animal re-ID. This collection provides well-structured metadata and is explicitly designed for benchmarking re-identification models under varying viewpoints and acquisition conditions. In parallel, the AnimalCLEF series^8^ has begun to include herpetological taxa in its multi-species individual ID challenges, most recently combining loggerhead sea turtles from an unreleased extension of SeaTurtleID2022 with a new dataset of *Salamandra salamandra*^8^, avialable via WildlifeDatasets^9^, for open-set recognition and long-term monitoring scenarios. Together, these resources demonstrate the feasibility of large-scale, open reptile and amphibian re-identification benchmarks, but they predominantly target aquatic species and facial or lateral views, leaving a gap for standardized, long-term datasets on small terrestrial lizards based on ventral scale patterns.

## Methods

### Dataset collection

The BalearicLizard dataset^6^ is derived from the long-term monitoring program of the Balearic wall lizard populations (*Podarcis lilfordi*) on multiple islets from the Balearic archipelago. For this Data Descriptor, we focus on a single, well-defined study site: Illot d’en Curt, a small rocky islet of approximately 4,394 m^2^ located off the southern coast of Mallorca^10^. The site hosts a dense, closed population of *P. lilfordi* that has been monitored annually since 2010.

Field surveys were conducted biannually (April and October) until 2019 and annually (October) from 2020 onwards, with each sampling season comprising three consecutive days of capture-mark-recapture surveys^11^. A grid of 26 georeferenced pitfall traps was deployed along paths and vegetation edges at an average spacing of approximately 3.5 m, covering an area of about 0.4 ha^12^.

Each captured lizard was photographed to enable subsequent individual identification based on the unique scale patterns in the pectoral and anterior ventral region^13,14^. While individuals were handled for photography, sex was determined by inspection of femoral pores and snout-to-vent length (SVL) and body mass were recorded.

**Fig. 2:**
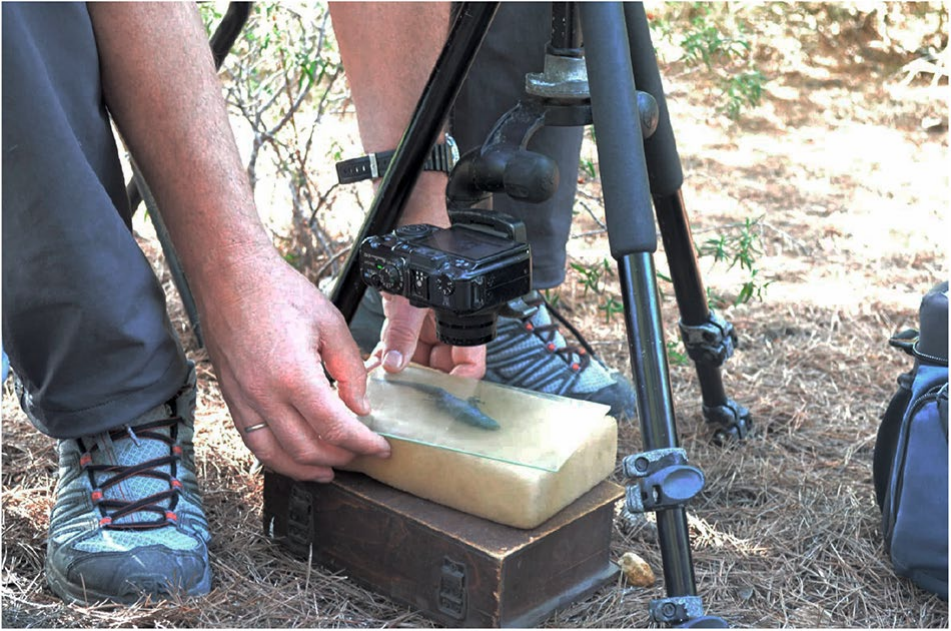
Photography set-up implemented for image acquisition during fieldwork.

Photographs were taken using digital DSLR or mirrorless cameras (various models; EXIF information retained in the released images) mounted on a tripod, as shown in Figure 2. A custom methacrylate frame with a sponge support was used to gently restrain the animal in a stable ventral-up position. This setup ensured that the ventral scales were centred in the frame, with the body approximately perpendicular to the camera sensor and minimal perspective distortion. Images were acquired under natural light conditions, without flash and without sedation. After measurement and photography, all individuals were released at their original capture locations.

Over 15 years (October 2010 to September 2024), this protocol yielded 4,619 usable images from 1,009 unique individuals across 73 field days. Only images that met basic quality standards (adequate focus, visibility of the scale region, and absence of severe occlusion) were retained for inclusion in BalearicLizard^6^; details of the quality checks and downstream corrections are provided in the Technical Validation section.

Although the photographic protocol has been described in the context of the ReMatch re-identification study^15^, we document the key details here to ensure that the dataset can be correctly interpreted and reused as a self-contained resource, as is standard practice in data descriptor publications.

### Data Preprocessing

The BalearicLizard^6^ repository distributes two main image products for each capture: (i) the original field photograph (after standard orientation) and (ii) a cropped image focusing on the biometric region. Here we describe the preprocessing steps applied to generate these products.

#### Initial standardisation

All raw images were first converted to a standard orientation using the EXIF rotation tags recorded by the camera. No further global adjustments were applied to the images prior to region of interest (in advance, ROI) extraction: in particular, we did not perform any colour correction, contrast stretching, denoising, or histogram equalisation. This decision preserves the natural variability in illumination and colour present in the field conditions while keeping the preprocessing pipeline simple and reproducible.

The resulting oriented images are stored in the images/ directory of the dataset as JPEG files, organised by individual identifier:

images/<individual_id>/<image_name>.jpg where <individual_id> is the curated individual code and <image_name> is the original filename.

#### Biometric region segmentation

To obtain standardised crops of the ventral pattern suitable for computer vision workflows, we applied a two-step segmentation pipeline as explained in the ReMatch study^15^. First, we used Grounded-SAM^16^ with text prompts targeting the central chest pattern (e.g. “central part of the chest scale pattern without head and arms”) to generate draft segmentation masks on a subset of images. These draft masks were inspected and manually refined when necessary to delineate the region containing the scales.

**Fig. 3:**
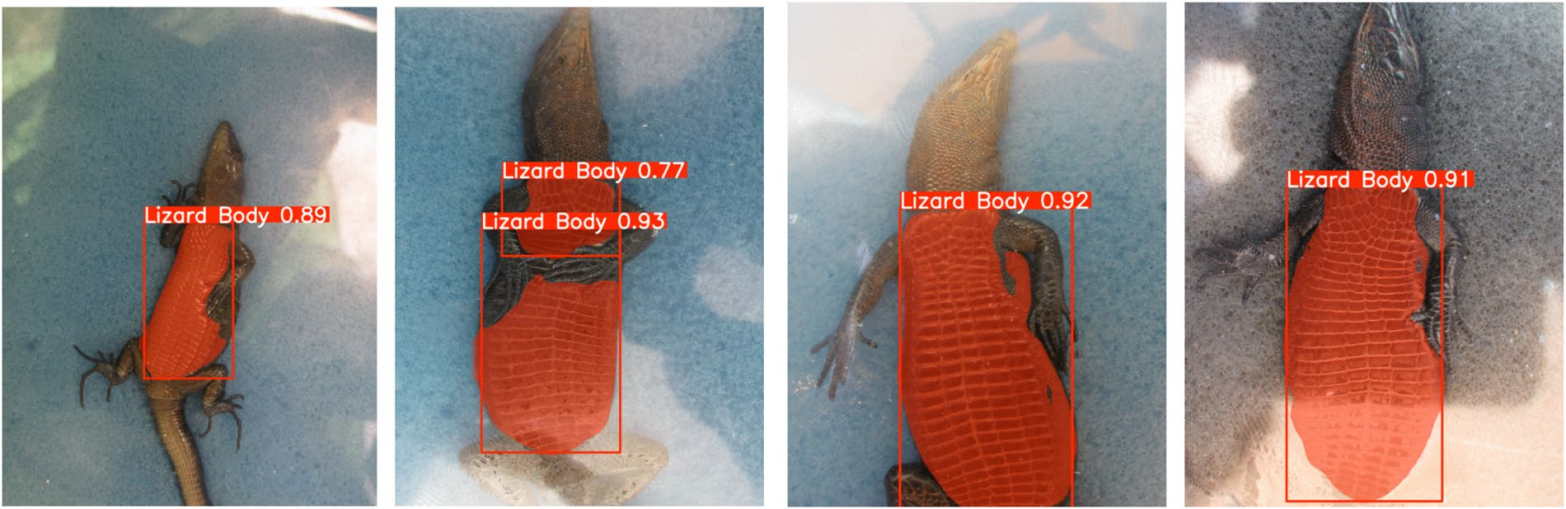
Examples of ventral-region detections from the YOLOv8 segmentation model. From left to right: (1) a low-confidence detection (score  < 0.9) that is automatically flagged for expert review; (2) an image with two overlapping detections, which is also flagged as ambiguous; (3–4) clean, high-confidence detections that pass automatic quality control and are used directly to generate the biometric crops stored in images-segmented/.

A YOLOv8^17^ segmentation model was then trained on 370 manually annotated examples to generalise the extraction of the biometric region across the full corpus. The model was trained to detect a single class corresponding to the ventral region of interest (ROI). On a held-out test set, the trained model achieved a precision of 0.9986, recall of 1.0, and mAP@50 of 0.995, indicating highly reliable segmentation performance. For the full dataset, detections with confidence  <0.9 or images with multiple overlapping masks were flagged for human inspection; images for which a satisfactory ROI could not be confirmed were excluded from the segmented subset. Some examples can be seen in Fig. 3.

For each successfully segmented image, we extracted the tight bounding box around the predicted ROI and saved the corresponding crop as a PNG file in the images-segmented/ directory:


images-segmented/<individual_id>/<image_name>.png


The file naming mirrors the original JPEGs, facilitating direct pairing between full-frame images and their associated biometric crops. Crops preserve the original pixel resolution within the bounding box; we did not rescale them to a fixed size in the released dataset, leaving downstream users free to choose their own resizing or normalisation strategies.

### Dataset Annotation

Individual identities in BalearicLizard^6^ are derived from the long-term capture–recapture work on Illot d’en Curt, using a semi-automated photo-identification workflow based on the APHIS software (Automated PHoto-Identification Suite)^18^. In this system, individual recognition relies on the unique arrangement of scales pattern in the ventral region of each lizard, which remains stable throughout an individual’s lifetime.

During the initial field sessions, newly captured lizards were assigned provisional individual identifiers and photographed under the standardised protocol described in the Dataset collection section. For subsequent captures, the new photograph was processed with APHIS^18^, which compares the ventral pattern of the “query” image against a library of previously catalogued images and returns a ranked list of candidate matches.

In practice, observers used the APHIS^18^ ranking as decision support: they visually inspected the top-ranked candidate images, comparing the pectoral scale pattern together with other stable morphological traits (e.g. head shape, relative size, scars or distinctive marks). When a clear match was found, the new capture was assigned to the corresponding individual ID in the catalogue. If no satisfactory match could be established among the candidates, a new individual ID was created and added to the reference library.

Finally, as part of the ReMatch^15^ study, we used AI automated re-identification models to screen for potential inconsistencies in the historical assignments. Candidate misidentifications flagged by the model were reviewed under expert supervision, leading to the correction of 94 individual IDs (more than 9% of the 1,009 final individuals). The identifiers and image-level annotations distributed in BalearicLizard^6^ therefore represent a curated and internally consistent version of the Illot d’en Curt catalogue, corrected for historical labelling errors.

For each image included in the dataset, the metadata file (metadata.csv) records the following variables: capture date, relative file path to the image, individual ID, and image filename. This lightweight annotation scheme is sufficient to support typical workflows in ecological demography and computer vision (e.g. individual re-identification, capture histories, and temporal patterns of re-sighting), while keeping the data format simple and easy to integrate into existing pipelines. Additional biological variables recorded during fieldwork (sex, snout-vent length, and body mass) were not consistently available across all capture events and are therefore not included in the current release. Detailed descriptions of the metadata fields are provided in the next section.

## Data Records

The BalearicLizard^6^ dataset comprises 4,619 standardised photographic images of captured Balearic wall lizards (*Podarcis lilfordi*) collected across 73 sampling days over 15 years (October 2010 to September 2024) from a single, well-defined study site: Illot d’en Curt, a small rocky islet located off the southern coast of Mallorca. In total, the archive documents 1,009 unique individuals from a closed, long-term monitored population. These images and their associated metadata are distributed at Kaggle^6^, in a simple folder structure designed to facilitate reuse in ecological and computer-vision workflows. It provides both the original field photographs (after standard orientation) and derived crops of the biometric region, together with a lightweight metadata table linking each image to an individual ID and capture date.

The public archive is organised as follows:

BalearicLizard/

      images/

             <individual_id>/<image_name>.jpg

             ...

      images-segmented/

             <individual_id>/<image_name>.png

             ...

curt_metadata.csv

The images/ directory contains the oriented field photographs as JPEG files, grouped by curated individual identifier (<individual_id>). Filenames (<image_name>) correspond to the original camera filenames, preserving any temporal or organisational structure they encode. The images-segmented/ directory mirrors this hierarchy and naming convention, but stores PNG crops corresponding to the ventral region of interest (ROI) for each image, as produced by the segmentation pipeline described in the Methods section. For a given row in the metadata table (see below), the pair images/<id>/<name>.jpg and images-segmented/<id>/<name>.png therefore refer to the full-frame image and its associated ROI crop for the same capture event.

The metadata file metadata.csv is a UTF-8 encoded, comma-separated text file that provides one record per original image. At minimum, it links each image to an individual identifier and capture date, and specifies the relative path and filename used in the directory structure above. This single table is sufficient to reconstruct basic capture histories, to form train/validation/test splits for re-identification models, or to integrate BalearicLizard^6^ with additional ecological or environmental datasets. A description of the metadata can be seen in Table 1.

**Table 1:** Schema of the metadata.csv file.

Field	Type	Description
date	string (YYYY-MM-DD)	Capture date of the photograph (local field date).
path	string	Relative path from the dataset root to the image file, typically images/<individual_id>/<image_name>.jpg.
id	string	Curated individual identifier for the lizard depicted in the image.
name	string	Image filename (basename with extension), shared between the original image and its segmented counterpart.

### Core summary

Images: 4,619; Individuals: 1,009; Sampling days: 73; Site: Illot d’en Curt; Licence: CC BY 4.0.

### Annotation and metadata schema

Additional biological variables collected during fieldwork (sex, SVL, body mass) were not consistently available for all capture events and are therefore not included in the current metadata table. These variables may be incorporated in future dataset updates once their standardisation across the full monitoring period have been verified.

## Technical Validation

Here we summarise the quality-control procedures applied during field sampling, image preprocessing, and annotation, together with additional checks performed using automated re-identification models.

### Image quality and consistency

During fieldwork, photographs were acquired under a standardised protocol using tripod-mounted cameras and a custom frame to position lizards in a stable ventral-up posture. Immediately after each session, images were screened by the field team to remove gross failures such as severe motion blur, out-of-focus shots, or cases where the ventral pattern was not visible. Only images that satisfied these basic quality criteria were retained for further processing.

In the preprocessing stage, all images were standardised to an upright orientation using their EXIF rotation tags. We then applied the segmentation pipeline described in the Methods section to extract the biometric region. For each image, we recorded the detector confidence and the number of predicted ROIs. Images with low-confidence detections or with multiple overlapping candidate masks were flagged and manually reviewed. Cases where a reliable ROI could not be confirmed were excluded from the segmented subset, ensuring that all crops in images-segmented/ contain clearly visible patterns.

To verify the internal consistency of the archive, we used automated scripts to check that every row of metadata.csv points to an existing image file in the images/ directory, that all filenames are unique within each individual, and that all individual identifiers referenced in the metadata correspond to a valid directory name. We also confirmed that the segmented images in images-segmented/ form a one-to-one subset of the original images, with matching filenames and individual IDs.

### Annotation consistency and individual IDs

Individual identities in BalearicLizard^6^ are based on a long-term catalogue maintained for the Illot d’en Curt population, built using expert visual matching of the scale pattern and associated morphological traits. To minimise observer error, candidate matches across years were reviewed by at least two experienced observers, and ambiguous cases were excluded from the catalogue. This procedure reduces the risk of conflating distinct individuals or of splitting a single individual into multiple IDs.

As part of the development of the ReMatch re-identification framework^15^, we performed an additional, model-based quality check on the historical annotations. Automated matching scores were used to flag suspicious pairs of images (either unusually dissimilar images assigned to the same ID, or very similar images assigned to different IDs), which were then re-examined manually. This process led to the correction of 94 individual IDs, corresponding to approximately 9% of the 1,009 final individuals, under expert supervision. The identifiers distributed with BalearicLizard^6^ therefore represent a curated, internally consistent version of the Illot d’en Curt catalogue that has been explicitly corrected for historical mislabelling.

### Segmentation performance

The YOLOv8 segmentation model used to extract the biometric region was trained and evaluated on manually annotated images (see Methods). On a held-out test set, the model achieved a precision of 0.9986, recall of 1.0, and mAP@50 of 0.995 for the ventral ROI class, indicating that false positives and missed detections are rare. For the full dataset, we combined these quantitative metrics with manual review of low-confidence or ambiguous cases, ensuring that the released ROI crops are accurate representations of the intended biometric region.

### Ethics approval and permits

Fieldwork complied with institutional and national guidelines. Authorization for capturing individuals in the field was provided yearly by the Government of Balearic Islands (Ref: CEP 06/2010-2021). Ethical approval was not required for this research. No animals were harmed.

## Usage Notes

### Getting started

Users can begin by loading the metadata.csv file and using the path, id and name fields to locate images. For each row, the full-frame JPEG and its corresponding biometric crop can be found at images/<id>/<name>.jpg and images-segmented/<id>/<name>.png, respectively. Both image products use standard formats and can be inspected or visualised with common image-processing libraries.

### Re-identification and ecological analyses

The dataset is suitable for training and evaluating individual re-identification models, including metric-learning approaches and deep learning-based pipelines. Because all individuals belong to a closed, long-term monitored population, the metadata can also be used to reconstruct capture histories and multi-year re-sighting records for demographic analyses.

### Generalisation limits

All images correspond to standardised ventral photographs of captured lizards from a single small islet population. As such, models trained on BalearicLizard may require additional adaptation before being applied to other species, sites or imaging protocols (e.g., unconstrained camera-trap imagery). Users should consider these limits when interpreting model performance or transferring trained models to other systems.

## Data Availability

The BalearicLizard dataset^6^ described in this Data Descriptor is publicly available at Kaggle (10.34740/kaggle/ds/8888731) under the CC BY 4.0 licence. The repository contains all images and the metadata.csv file described in the Data Records section; no controlled-access restrictions apply.

## Code availability

All code used to preprocess the images is publicly available at https://github.com/RoberAlcaraz/ReMatch. Thecomplete re-identification pipeline will be madefully available in the same repository upon publication of the associated ReMatch study^7^.
